# Supplementary material for: Hyperacetylated chromatin domains mark cell type-specific genes and suggest distinct modes of enhancer function
Source: Nat Commun. 2020 Sep 11;11:4544. doi: 10.1038/s41467-020-18303-0 (PMC7486385; doi:10.1038/s41467-020-18303-0)
Supplement: Supplementary file 1 — Supplementary Information [file 41467_2020_18303_MOESM1_ESM.pdf]

Hyperacetylated Chromatin Domains Mark Cell Type-Specific Genes and Suggest Distinct Modes of Enhancer Function

Fox et al.

Supplementary Information

Supplementary Figure 1. Identification of hyperacetylated chromatin domains (HCDs) and super-enhancers.
Supplementary Figure 2. ChIP-seq profiles at selected gene loci in human erythroid cells.
Supplementary Figure 3. ChIP-seq profiles at selected gene loci in mouse intestinal epithelial cells.
Supplementary Figure 4. ChIP-seq profiles at selected gene loci in mouse retinal cells.
Supplementary Figure 5. Comparison of genes associated with super-enhancers vs. HCDs in murine intestinal epithelial cells.
Supplementary Figure 6. Comparison of genes associated with super-enhancers vs. HCDs in murine retinal cells.
Supplementary Figure 7. Comparison of genes associated with Me3 domains vs. HCDs in murine erythroid cells.
Supplementary Figure 8. Comparison of genes associated with different HCD identification methods in murine erythroid cells.
Supplementary Figure 9. Comparison of genes associated with different HCD identification methods in murine retinal cells.
Supplementary Figure 10. Effects of deletions of putative enhancers in the *SLC4A1* gene locus in differentiating MEL cells.
Supplementary Figure 11. Gating strategy for proerythroblasts.

Supplementary Table 1. sgRNAs for enhancer deletions and insertions
Supplementary Table 2. ChIP Primers
Supplementary Table 3. Expression Primers

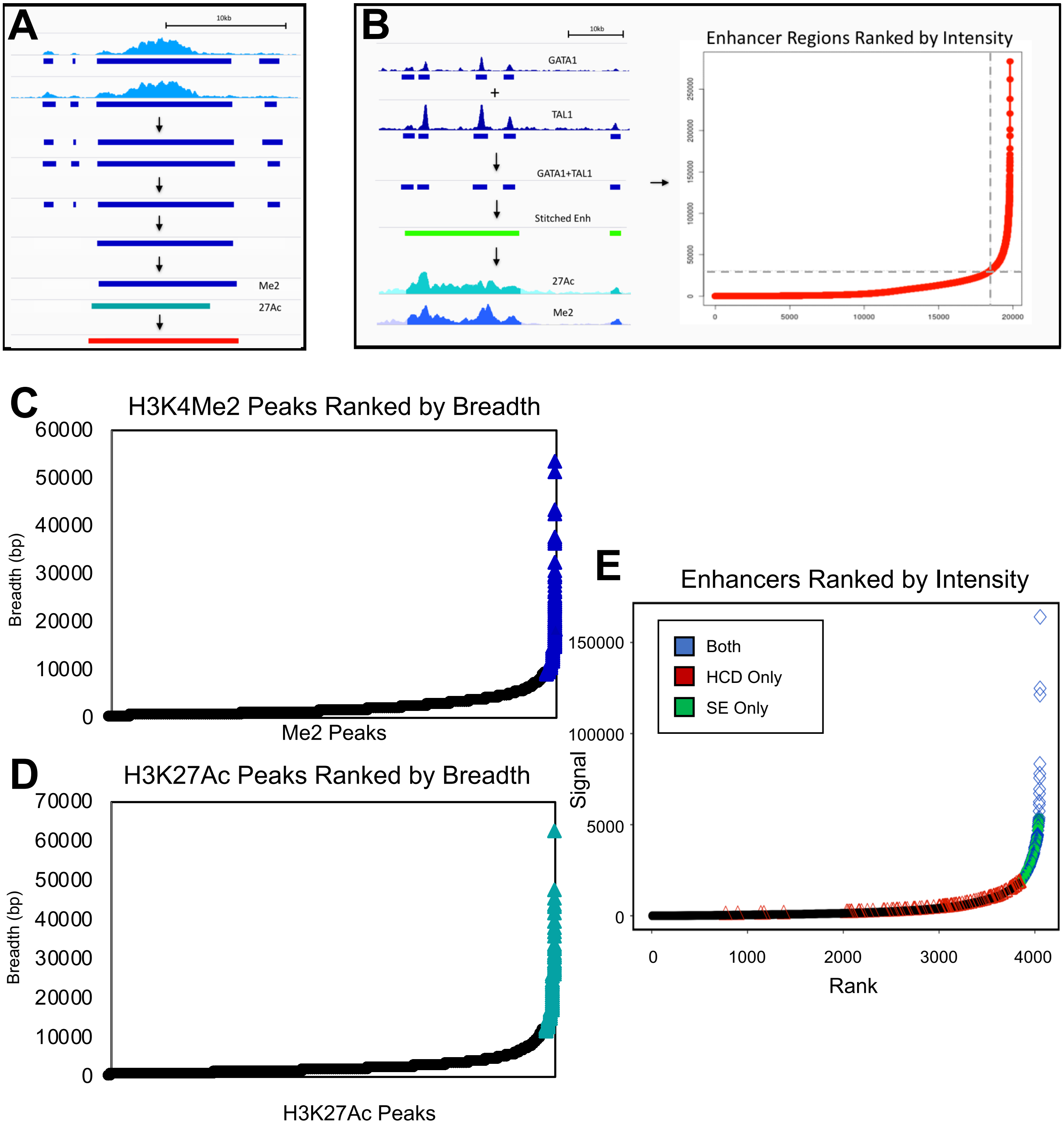

Supplementary Figure 1: Identification of hyperacetylated chromatin domains (HCDs) and super-enhancers. (A) Flow chart for HCD calls. MACS2 was used to call peaks in ChIP-seq tracks for H3K4Me2 and H3K27Ac. For each modification, 2 replicate ChIP-seqs were performed, and so the intersection of MACS2-called peaks was taken and then filtered for the top 2% broadest regions. The final list of HCDs was defined as the union of top 2% peaks that overlapped for each modification. **(B)** Flow chart and signal strength ranking graph for super-enhancer calls. A population of putative enhancers was called based on the union of MACS2-called peaks from GATA1 and TAL1 ChIP-seqs. The resulting list was then input into the ROSE algorithm with a stitching distance of 12.5 kb and an exclusion of +/- 500 bp around known TSSs, and ranking based on H3K27Ac reads from our own ChIP-seqs at the selected regions. **(C)** Graph of all H3K4Me2 peaks as ranked by breadth, with K4Me2 domains highlighted in blue. **(D)** Graph of all H3K27Ac peaks as ranked by breadth, with K27Ac domains highlighted in aqua. **(E)** Graph of all H3K27Ac peaks as ranked by ROSE, with super-enhancers highlighted in green, HCDs in red and regions called as both HCDs and super-enhancers in blue.

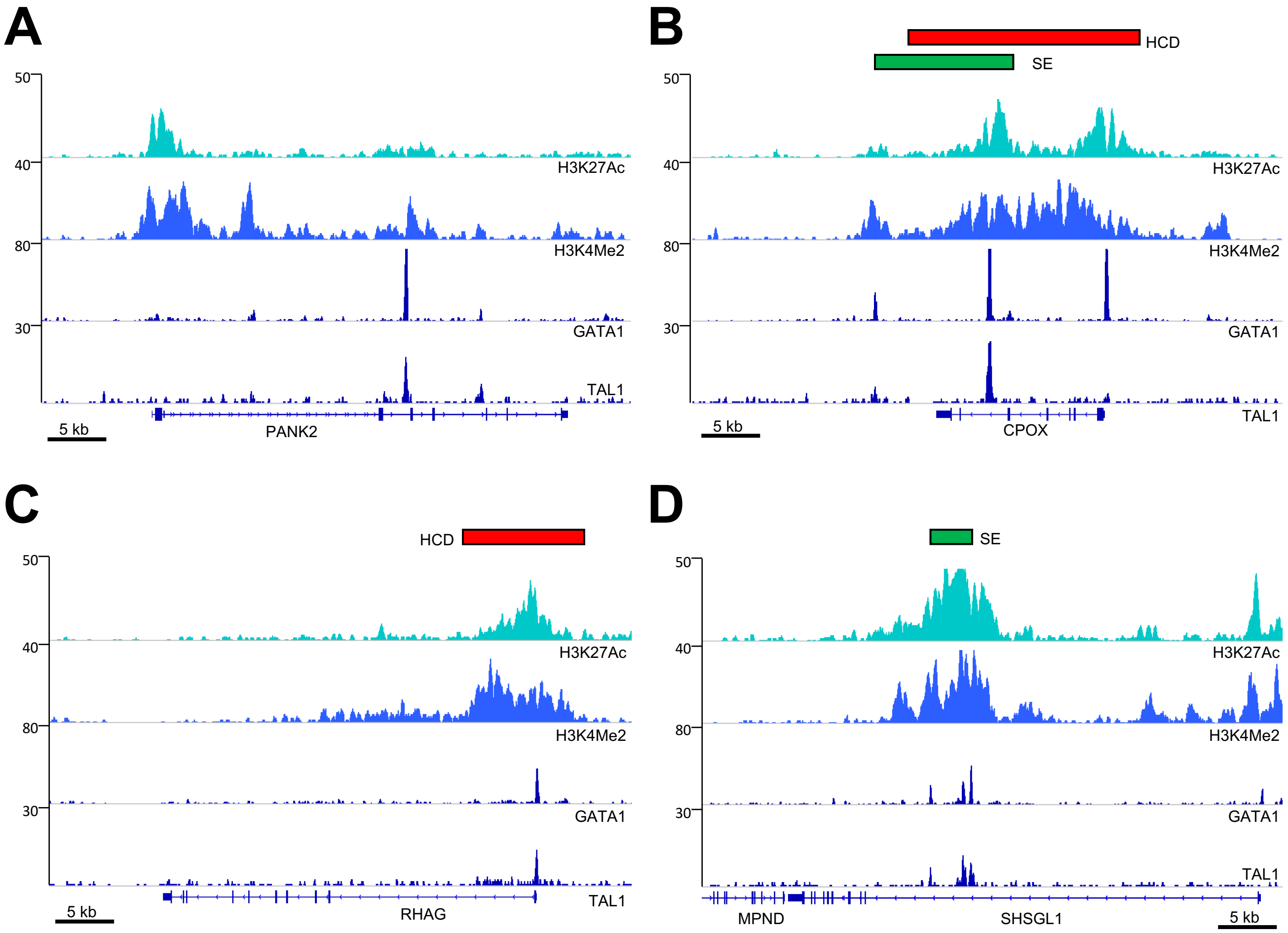

Supplementary Figure 2: ChIP-seq profiles at selected gene loci in human erythroid cells. Tracks show read densities for the indicated histone modifications or transcription factors. Genes and scale are shown at the bottom, and peak calls for super-enhancers and/or hyperacetylated chromatin domains (HCDs) at the top. **(A)** The P*ANK2* locus, harboring a putative enhancer that is called neither a super-enhancer nor an HCD. **(B)** The *CPOX* locus, harboring both a super-enhancer and an HCD. **(C)** The *RHAG* locus, harboring an HCD but not a super-enhancer. **(D)** The *SHSGL1* locus, harboring a super-enhancer but not an HCD.

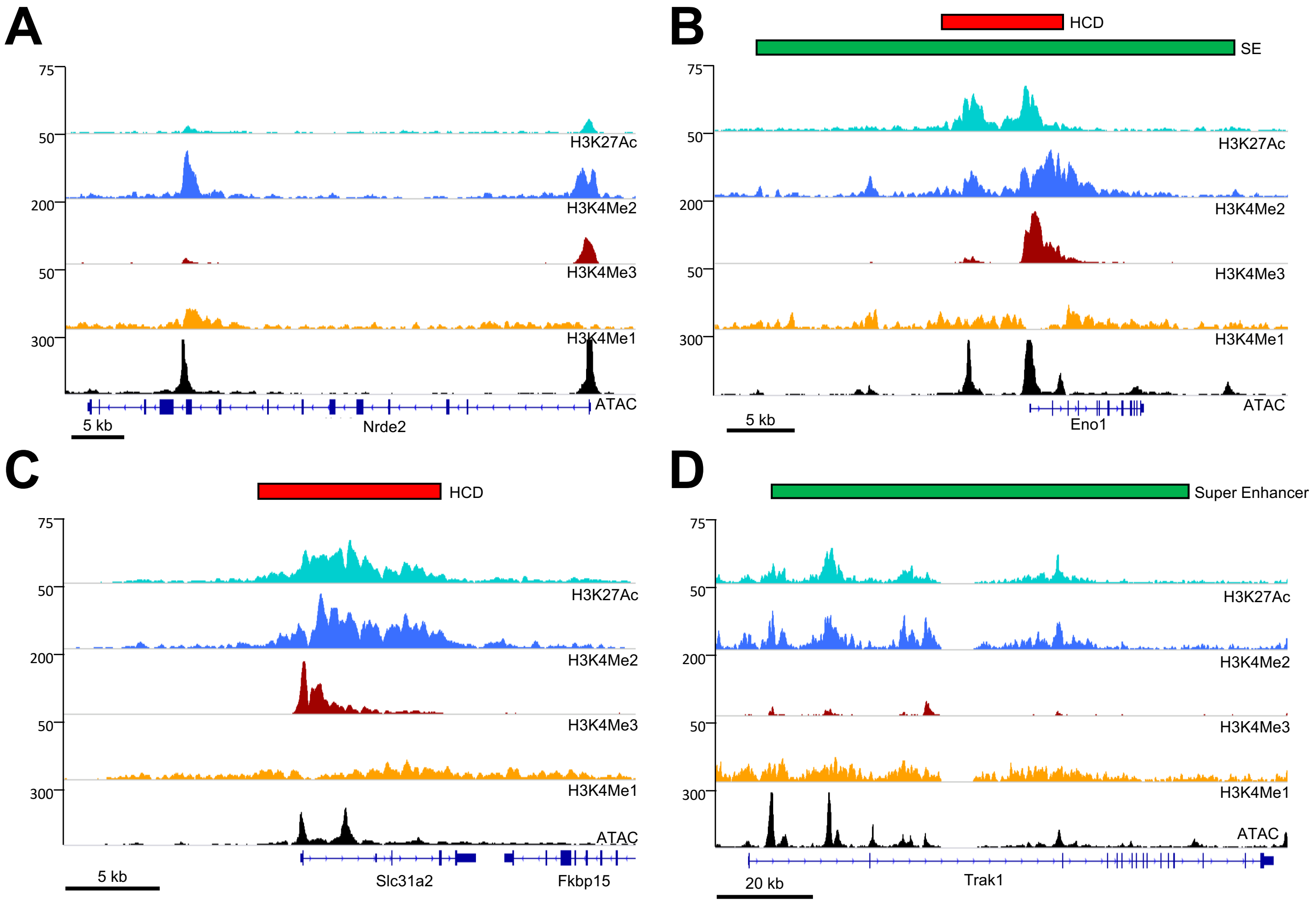

Supplementary Figure 3: ChIP-seq profiles at selected gene loci in mouse intestinal epithelial cells. Tracks show read densities for the indicated histone modifications or ATAC-seq. Genes and scale are shown at the bottom, and peak calls for super-enhancers and/or hyperacetylated chromatin domains (HCDs) at the top. **(A)** The *Abcc2* locus, harboring a putative enhancer that is called neither a super-enhancer nor an HCD. **(B)** The *Myh14* locus, harboring both a super-enhancer and an HCD. **(C)** The *Vil1* locus, harboring an HCD but not a super-enhancer. **(D)** The *Sis* locus, harboring a super-enhancer but not an HCD.

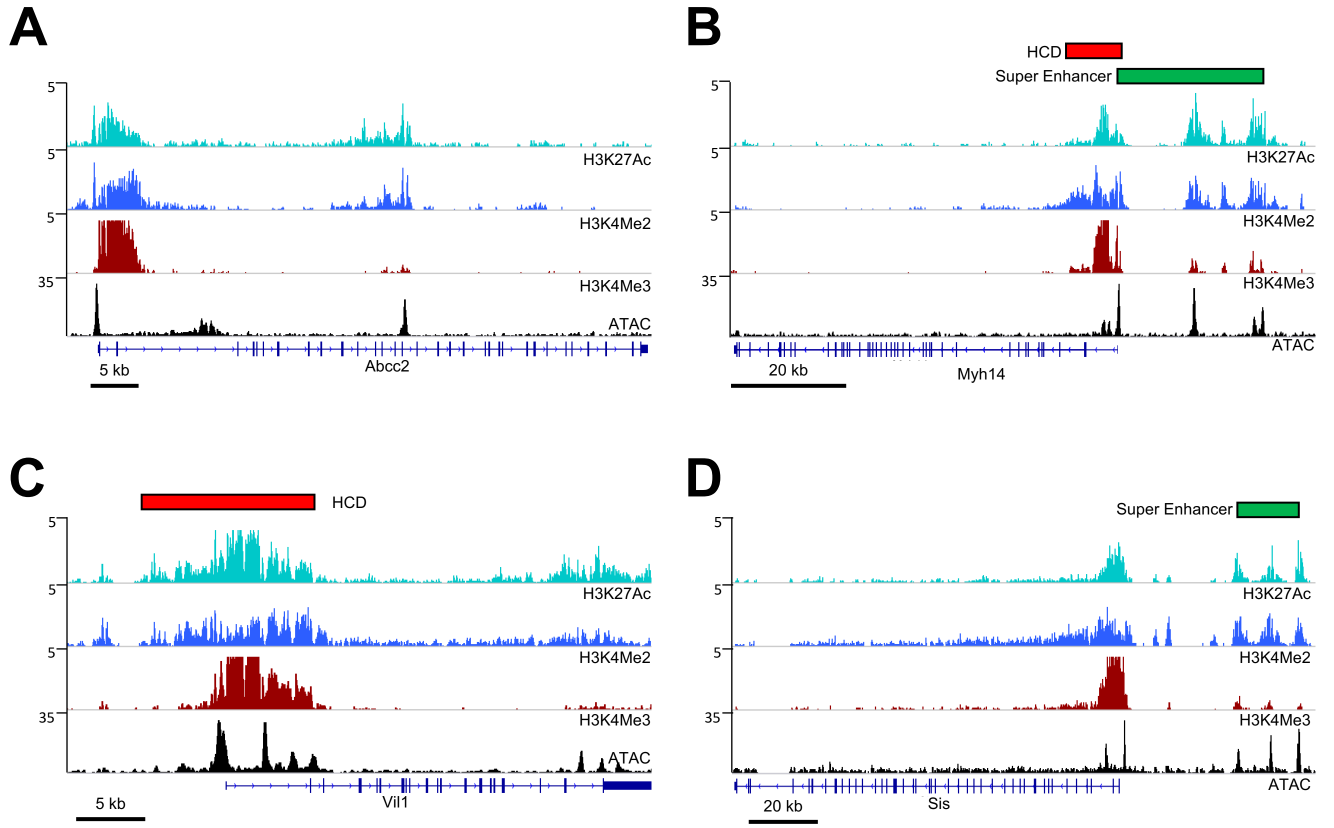

Supplementary Figure 4: ChIP-seq profiles at selected gene loci in mouse retinal cells. Tracks show read densities for the indicated histone modifications or ATAC-seq. Genes and scale are shown at the bottom, and peak calls for super-enhancers and/or hyperacetylated chromatin domains (HCDs) at the top. **(A)** The *Nrde2* locus, harboring a putative enhancer that is called neither a super-enhancer nor an HCD. **(B)** The *Eno1* locus, harboring both a super-enhancer and an HCD. **(C)** The *Slc31a2/Fkbp25* locus, harboring an HCD but not a super-enhancer. **(D)** The *Trak1* locus, harboring a super-enhancer but not an HCD.

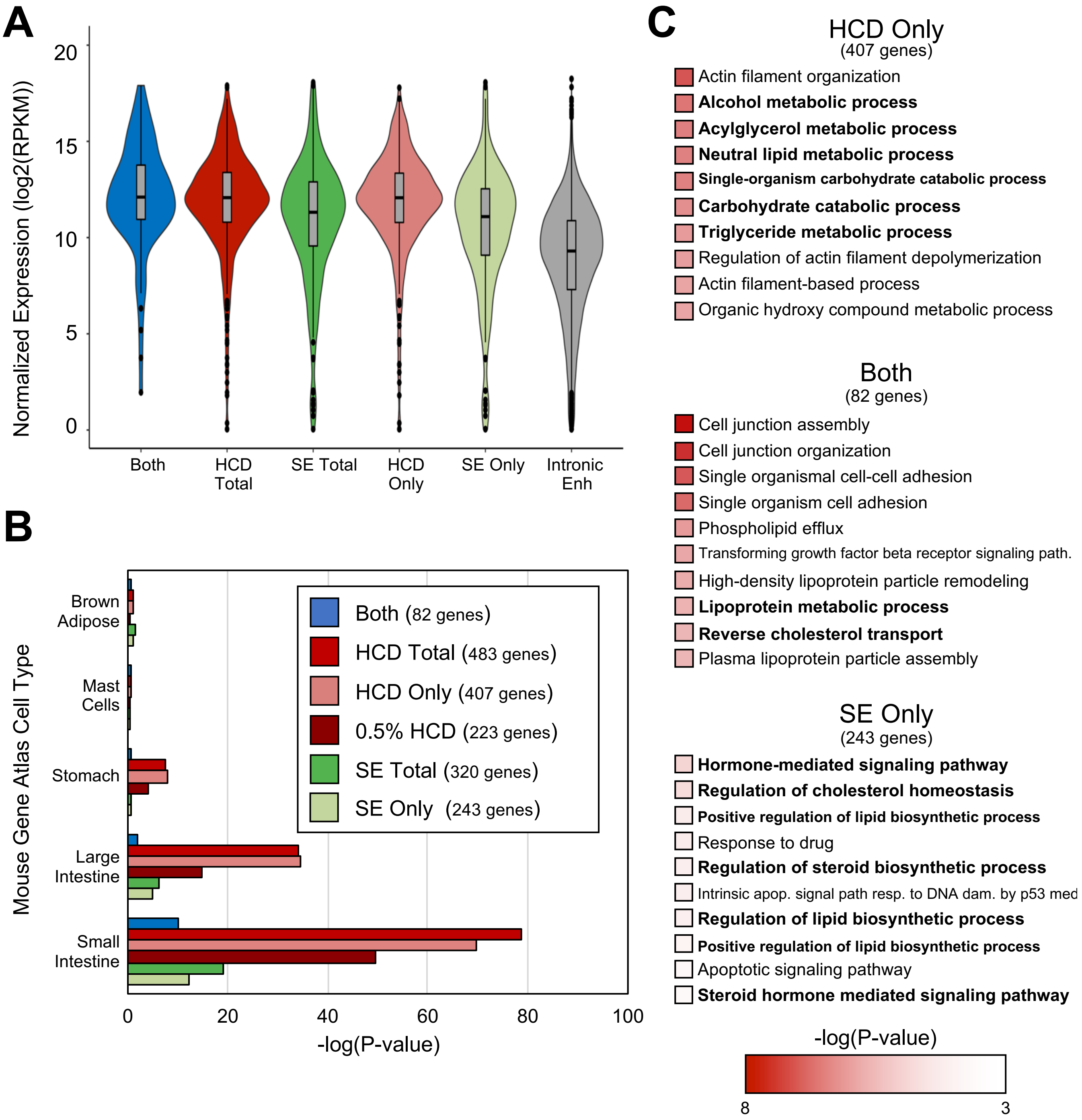

Supplementary Figure 5: Comparison of genes associated with super-enhancers vs. HCDs in murine intestinal epithelial cells. (A) Violin plots of expression of genes associated with HCDs, super-enhancers or both. Expression for genes associated with all putative enhancers located within introns is shown for comparison. In box plots, the center line represents the median, the box limits represent the 25th and 75th percentiles and the whiskers represent 1.5 times the interquartile range. **(B)** Bar graph showing P-value for Enrichr cell-type enrichment for the 5 cell types with the highest scores for each category. **(C)** Listings of the top ten GO terms for biological processes for the indicated groups; intestine-specific terms are in boldface type.

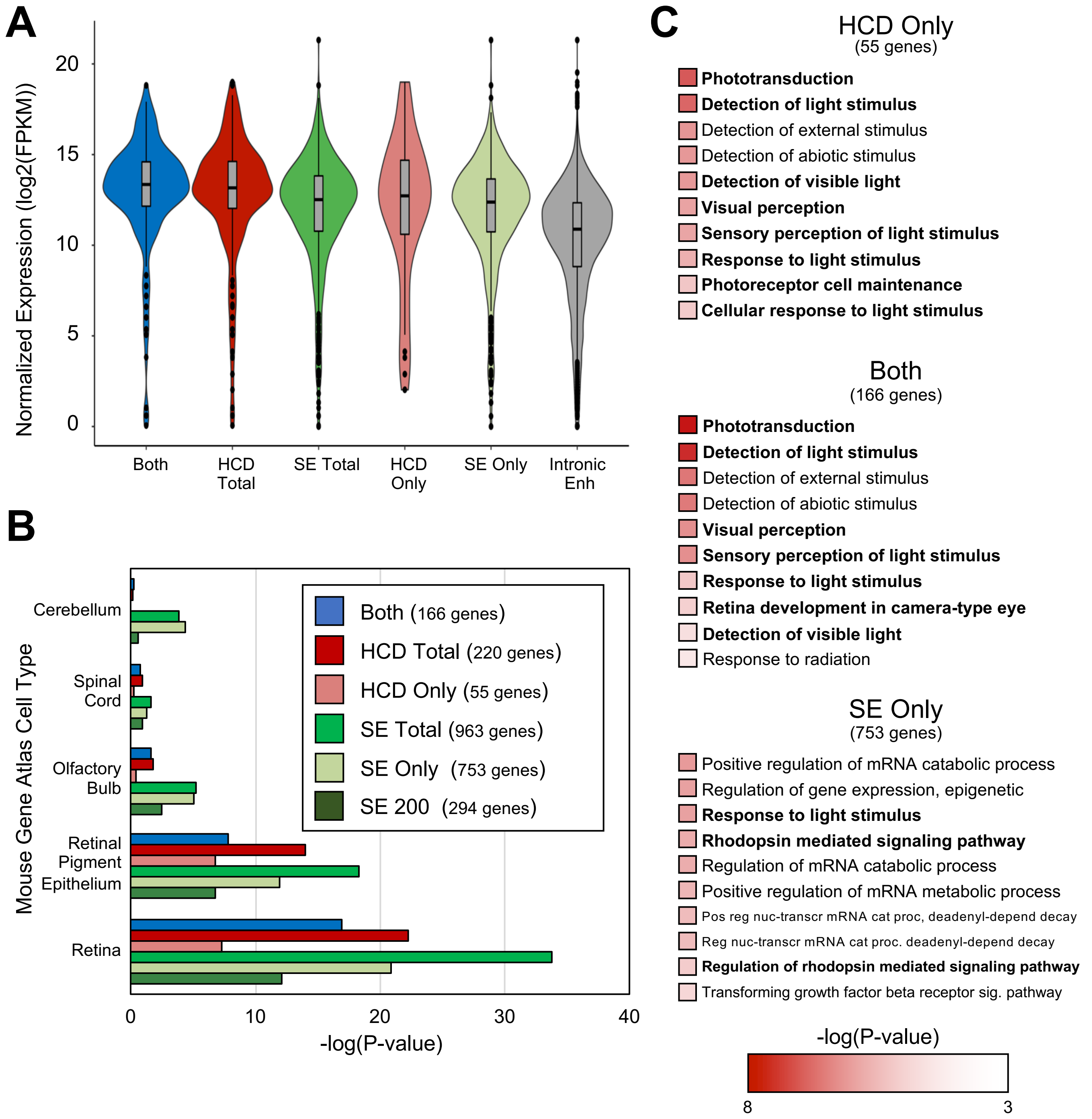

Supplementary Figure 6: Comparison of genes associated with super-enhancers vs. HCDs in murine retinal cells. **(A)** Violin plots of expression of genes associated with HCDs, super-enhancers or both. Expression for genes associated with all putative enhancers located within introns is shown for comparison. In box plots, the center line represents the median, the box limits represent the 25th and 75th percentiles and the whiskers represent 1.5 times the interquartile range. **(B)** Bar graph showing P-value for Enrichr cell-type enrichment for the 5 cell types with the highest scores for each category. **(C)** Listings of the top ten GO terms for biological processes for the indicated groups; retina-specific terms are in boldface type.

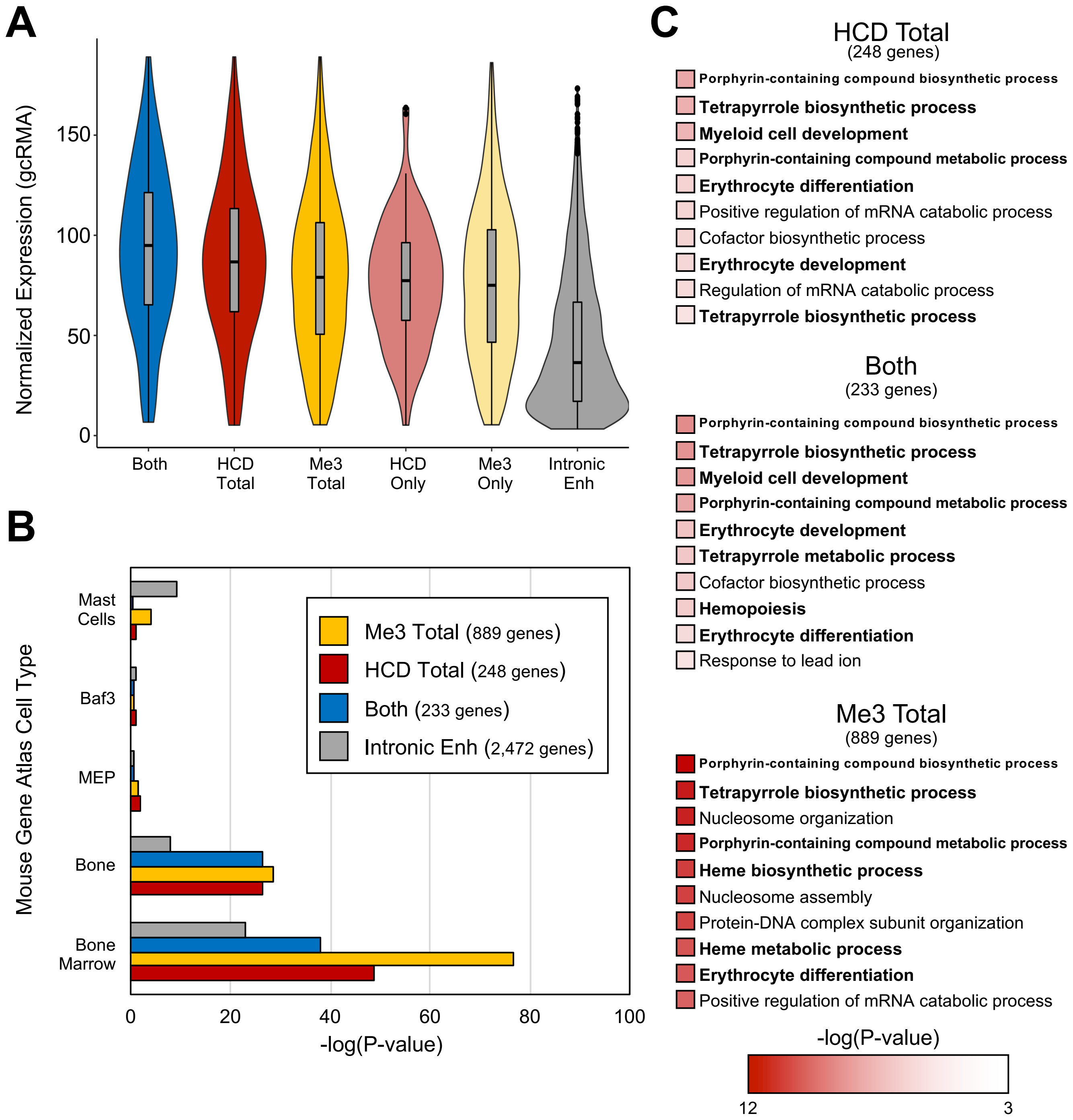

Supplementary Figure 7: Comparison of genes associated with Me3 domains vs. HCDs in murine erythroid cells. **(A)** Violin plots of expression of genes associated with HCDs, Me3 domains or both. Expression for genes associated with all putative enhancers located within introns is shown for comparison. In box plots, the center line represents the median, the box limits represent the 25th and 75th percentiles and the whiskers represent 1.5 times the interquartile range. **(B)** Bar graph showing P-value for Enrichr cell-type enrichment for the 5 cell types with the highest scores for each category. **(C)** Listings of the top ten GO terms for biological processes for the indicated groups; erythroid-specific terms are in boldface type.

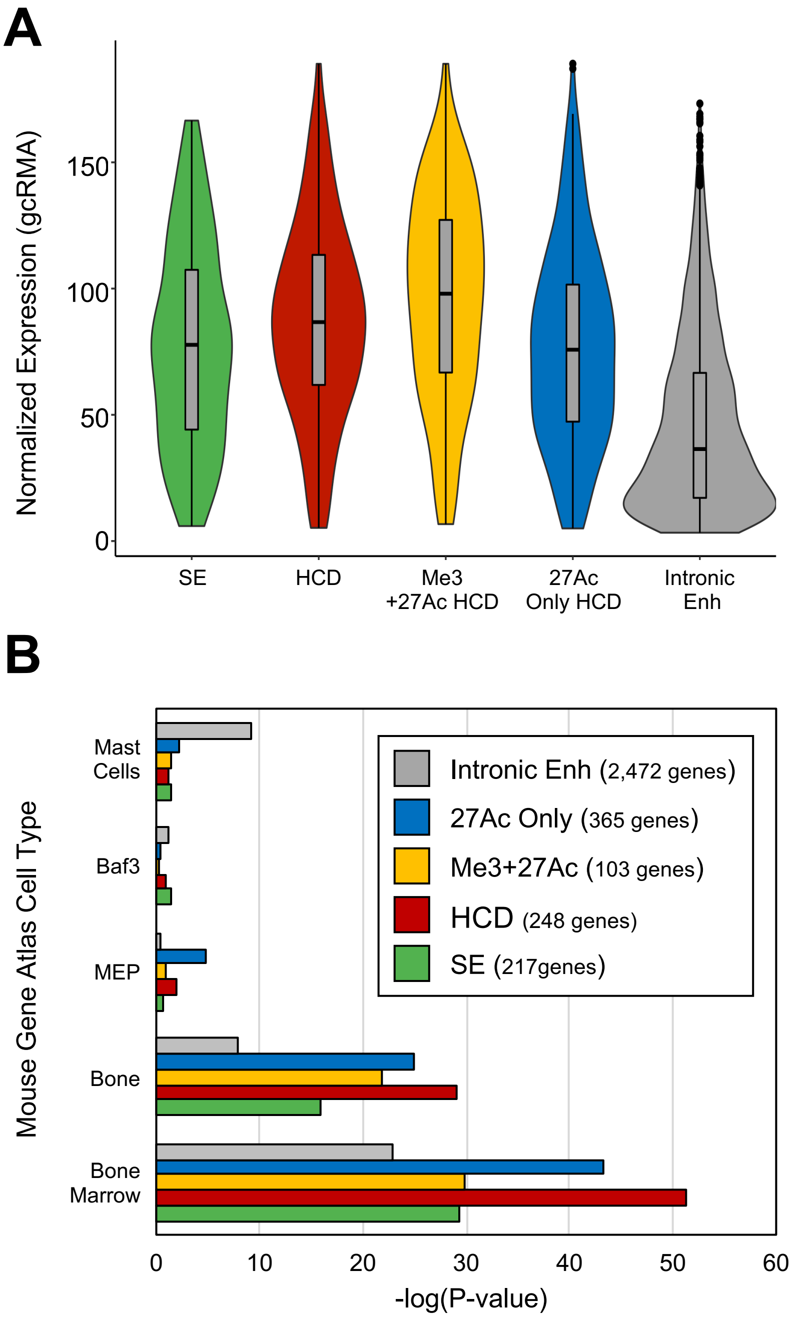

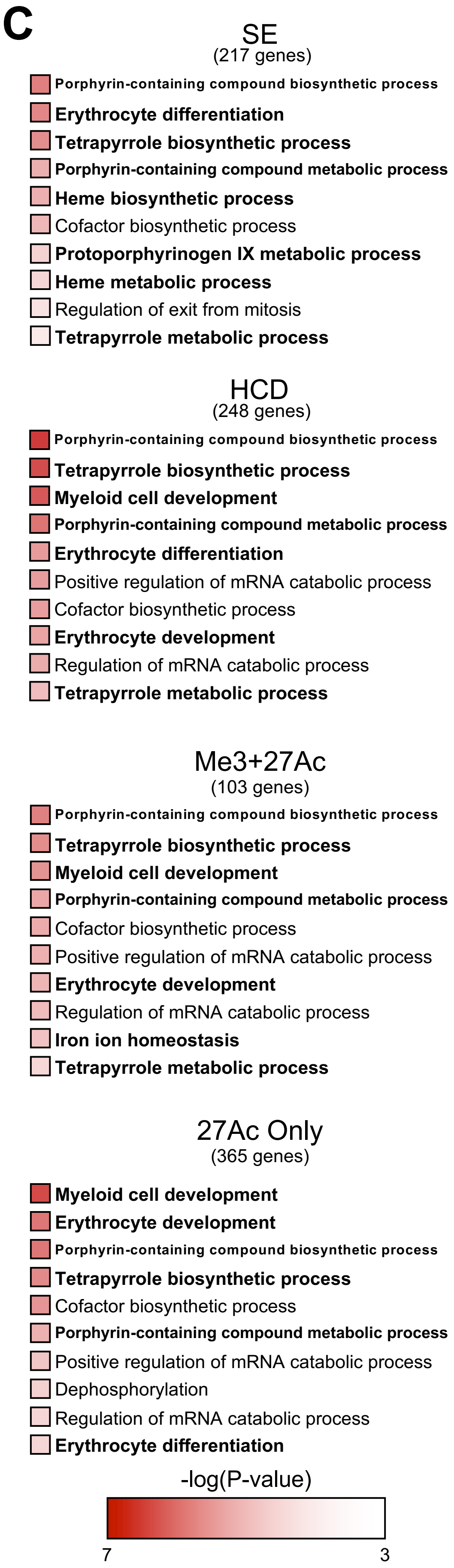

Supplementary Figure 8: Comparison of genes associated with different HCD identification methods in murine erythroid cells. **(A)** Violin plots of expression of genes associated with SEs, HCDs, Me3+27Ac HCDs, or 27Ac only HCDs. Expression for genes associated with all putative enhancers located within introns is shown for comparison. In box plots, the center line represents the median, the box limits represent the 25th and 75th percentiles and the whiskers represent 1.5 times the interquartile range. **(B)** Bar graph showing P-value for Enrichr cell-type enrichment for the 5 cell types with the highest scores for each category. **(C)** Listings of the top ten GO terms for biological processes for the indicated groups; erythroid-specific terms are in boldface type.

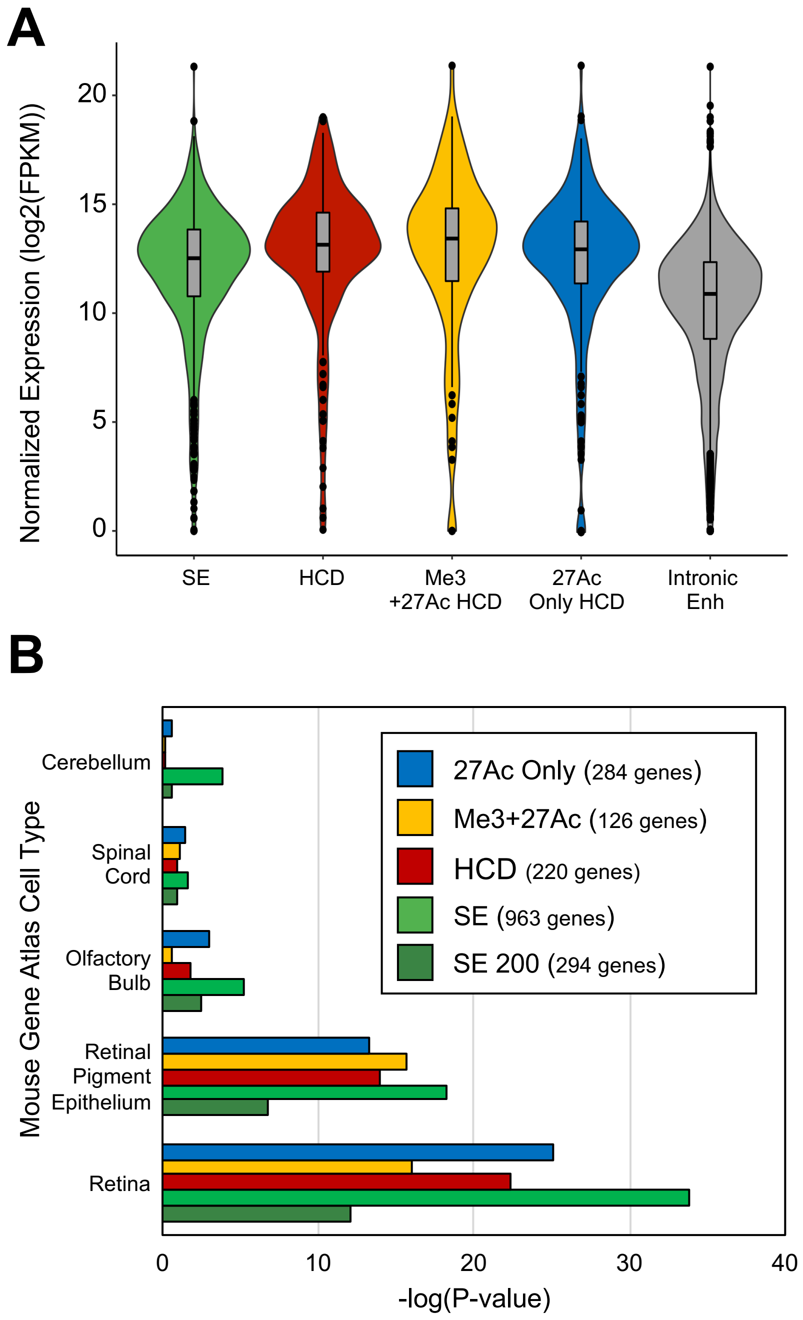

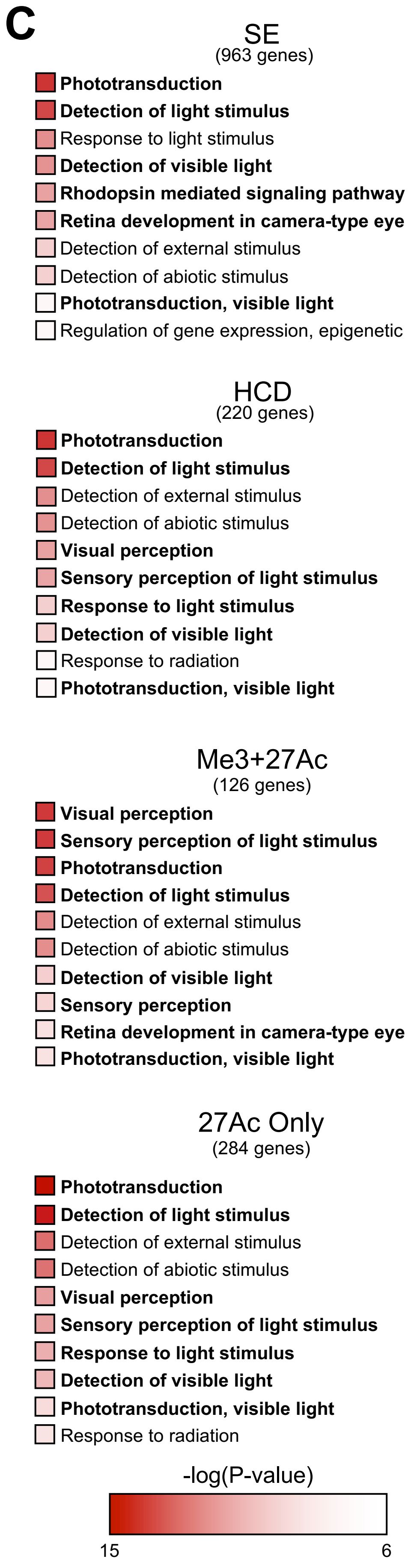

Supplementary Figure 9: Comparison of genes associated with different HCD identification methods in murine retinal cells. **(A)** Violin plots of expression of genes associated with SEs, HCDs, Me3+27Ac HCDs, or 27Ac only HCDs. Expression for genes associated with all putative enhancers located within introns is shown for comparison. In box plots, the center line represents the median, the box limits represent the 25th and 75th percentiles and the whiskers represent 1.5 times the interquartile range. **(B)** Bar graph showing P-value for Enrichr cell-type enrichment for the 5 cell types with the highest scores for each category. **(C)** Listings of the top ten GO terms for biological processes for the indicated groups; retina-specific terms are in boldface type.

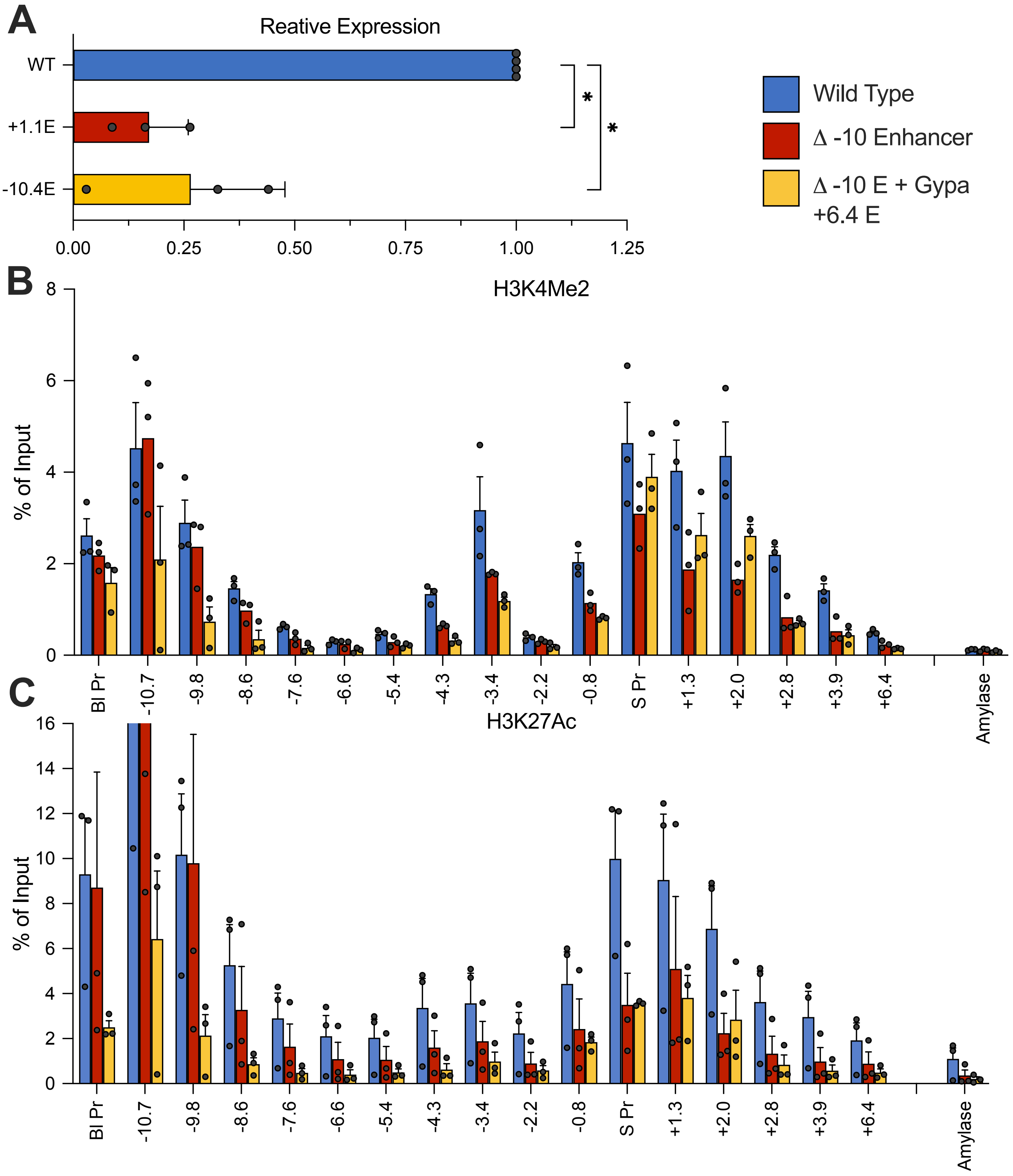

Supplementary Figure 10: Effects of deletions of putative enhancers in the Band3 gene locus in differentiating MEL cells. (A) Normalized gene expression for Band3 measured by qrt-PCR in wt (MEL) and upon deletion of the indicated enhancer regions (+1.1 or -10.4). P-values were calculated using a two-tailed Student's t-test; * = P<0.05. Results are means + s.e.m. **(B+C)** Bar graph showing percent of input control obtained using PCR probes at the indicated locations (in kb) relative to the transcription start site for the Band3 gene in ChIP assays using antibodies specific for H3K4Me2 **(B)** or H3K27Ac **(C)** "Amylase" indicates a control probe within the inactive amylase gene locus. Results of all panels are means + s.e.m of at least 3 independent experiments. Each colored circle represents the average of 3 technical replicates of an independent single-cell-derived homozygous enhancer knockout clone.

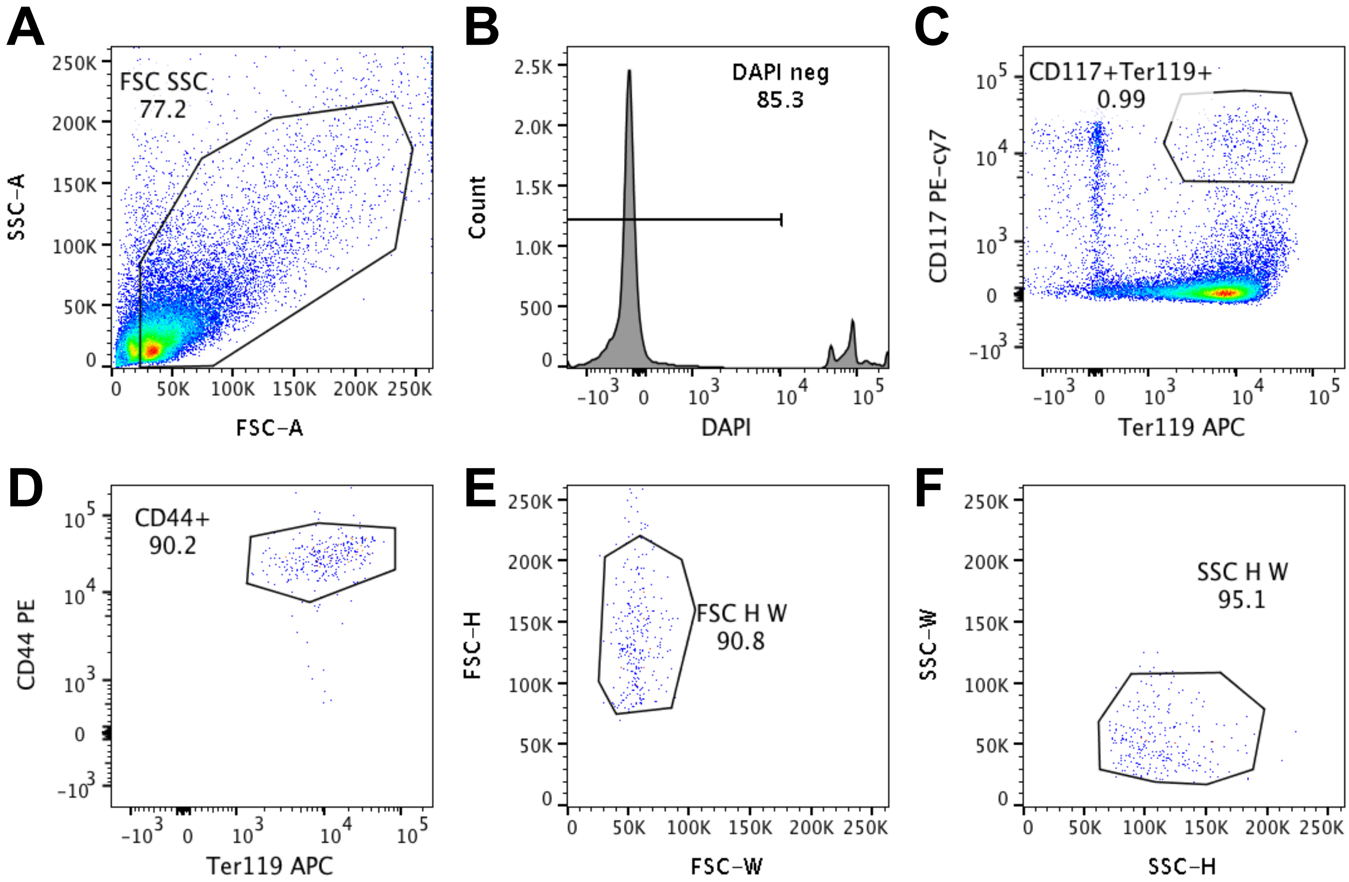

Supplementary Figure 11: Gating strategy for proerythroblasts. Proerythroblasts were sorted initially using **(A)** a broad FSC-A/SSC-A size gate containing ~80% of the cells. Following gating for **(B)** live cells **(C)** CD117+ Ter+ double positive cells (1-2%) were **(D)** further confirmed to be CD44+ (90%), and singlets were obtained by **(E)** FSCH/W and **(F)** SSC H/W gating.

Region	Targeted DNA 5’ to 3’
Gypa +6.4 FWD	GCCATATCTTGATTGCGTAATGG
Gypa +6.4 REV	ACCAGACTTAGACTGCATGGAGG
Gypa Pr FWD	TAGGAGTGTTTCTAATGCGGGGG
Gypa Pr FREV	ACTATGAATTGCCCCAATGCTGG
Tspan32 -17 FWD	GAGGGATTAAGTAGACGTCAAGG
Tspan32 -17 REV	AAATTGCATCAGTTTGGCTTTGG
Tspan32 -10 FWD	GATGGCATGACTACCTAGCCAGG
Tspan32 -10 REV	GACTCTTAGGAAACCTGAACTGG
Tspan32 +0.7 FWD	CAGGGAATACCATATACAGAGGG
Tspan32 +0.7 REV	AGCCTGGGGACTGGGCATAGAGG
Band3 +1.1 FWD	GCTCTTAGCTGATTGGGAGTTGG
Band3 +1.1 REV	TGGAACTCACTCTGTAGACCAGG
Band3 -10.4 FWD	CTTGAACCTTTAGAGACTTCTGG
Band3 -10.4 REV	AACCTAGGGGATTCCAAGGAAGG
Tspan32 -10.0 IN	GTGAGTGCTGCCCGTGGGCTGG

Supplementary Table 1. sgRNAs for enhancer deletions and insertions

Primer	Sequence 5’ to 3’
Gypa -3.0 FWD	ACG CAG TAT CTT TGG TGA AGG AAC A
Gypa-3.0 REV	GTT CAT ACC TTA CCT TGT GGC CTC T
Gypa -1.8 FWD	GAC CCC TCC CTC CGT TCT TCC
Gypa -1.8 REV	TGT GGT TCT GAC AGG CTT CAA CTC
Gypa -0.94 FWD	ACC TGA TGA TAC TCC TGG ACA ACG
Gypa -0.94 REV	GGC GAC CTC TAG TGG CTG TTC
Gypa Pr FWD	GCC AAT GTG TGT CAA CGG ATA GAG
Gypa Pr REV	GGA GTG TCT GTC AGC CTT AGG AAG
Gypa +0.6 FWD	AGG GGT TTC TCT TTG TGG AAC TGT
Gypa +0.6 REV	ATT CAG CCC AGC CAT AGA GCA ATT
Gypa +1.73 FWD	CGT AAT AAC CGC ATA AGG GCT GAG
Gypa +1.73 REV	CGG GTG GAC TGA AAT ACA AGG C
Gypa +2.7 FWD	CAC ACA GAA GCA AAC ACT CAT CCC
Gypa +2.7 REV	CTG CCA TCT CAC CTC CAG TTA CG
Gypa +3.2 FWD	AAG CCT ATC CCG AGC AGA GC
Gypa +3.2 REV	TGA TTC TAA CGA CCA CAG CAC AG
Gypa +5.0 FWD	TCT CAC ACT CAA CCG TAT TCC
Gypa +5.0 REV	CTT CAC TAA TGG CTT CAG TTA GC
Gypa +6.0 FWD	GTG CCT TGA GTA AAC TTC CAG TGT C
Gypa +6.0 REV	CTC TGC CTA GCT GTC TCG TCT ATG
Gypa +8.5 FWD	GAG GGA GCC ATT AAG ACG TGA CAC
Gypa +8.5 REV	ACT TGC TTC TTG GTG GTG GAA CTC
Tspan32 -18.5 FWD	GGT CTT TAA GAT TCA GAG CTA GTG C
Tspan32 -18.5 REV	CAG GTT GGC CTC AAA CTC C
Tspan32 -11.5 FWD	GCT TTA TGT GCA TGT ATG TGT GC
Tspan32 -11.5 REV	GAC TCA TCG TCA TCA GTA ACT CC
Tspan32 -10.4 FWD	CTA GAG AAT TCA CTG GTC ACT AGG
Tspan32 -10.4 REV	GTG ACT AAT GGC TGT GGT AGG
Tspan32 -9.4 FWD	ACA GTG TGG AGG TAG AGA GG
Tspan32 -9.4 REV	TCC CTT CTG GGA GTG AGG
Tspan32 -8.3 FWD	CTC CCA ACC TAT CTT CAA CTT CC
Tspan32 -8.3 REV	TCA TGC ATT AGC TAG TGG TTC C
Tspan32 -7.3 FWD	CAC TGT CCA ATA CAC AAA GAG C
Tspan32 -7.3 REV	GAA GAG AAC AGA TCT CCC ATC C
Tspan32 -6.0 FWD	GCA CAC ATA CAC ACA ATC TTC C
Tspan32 -6.0 REV	AAT GGT AAT GTT GTT CCC TTT CC
Tspan32 -5.1 FWD	GGC CTT CTT TCT GGT TTA TAT TTA GG
Tspan32 -5.1 REV	ATG CTC AGT GCT TTA ATT GTG G
Tspan32 -3.8 FWD	TCC AAC CAC TTA ACC ATT CTC C
Tspan32 -3.8 REV	ACT GGG ATA TAC CTT CCT TTG C
Tspan32 -3.1 FWD	GCA GGG CTA CTC AAC AAA CC
Tspan32 -3.1 REV	TCT GTG ACT GTG TGA TGT TTG G
Tspan32 -2.0 FWD	TAC TCT CCA AAG CCC ACT TAG C
Tspan32 -2.0 REV	GCG TCT GCA ATA ACA CAC AGG

Supplementary Table 2. ChIP Primers

Primer	Sequence 5' to 3'
Tspan32 -1.1 FWD	CCA TAG GTC CTT CTC TTT CTG C
Tspan32 -1.1 REV	CTA TAC CAG CTG TTG CTC TGG
Tspan32 Pr FWD	GGA GAC CAC TTT ACT GTG ATT GG
Tspan32 Pr REV	GTT CCT CCC AGA TGT GTT TGG
Tspan32 +1.2 FWD	AGA GCT GGA GAG GAA TAA GAC C
Tspan32 +1.2 REV	CCT GAT TGA GCA AGT GGA AGG
Tspan32 +2.2 FWD	AGG CTG GCC ATG AAT AAG C
Tspan32 +2.2 REV	TTT GTC TGT CCT ACA TGC TTC C
Tspan32 +3.3 FWD	GGA ATG GCT TCC AGA TGG
Tspan32 +3.3 REV	CCT TCT CCT TCT CTC CTC TAC C
Tspan32 +4.4 FWD	TGA GTC AGG CTA TAG AGC TTA GG
Tspan32 +4.4 REV	CAA ACT TCT CTC TTG AAG GTA GGG
Bloodlinc Pr FWD	CTG GAA CTG GAG TTG TAG ATA GG
Bloodlinc Pr REV	GAA AGT CAG GAT GGA GTC TGG
Band3 -10.7 FWD	CAC AGG GAC TGA AGG AAC G
Band3 -10.7 REV	TGT GCC CAT CTC AAT CGC
Band3 -9.8 FWD	CTC CAA GGT CCA CAC AAG G
Band3 -9.8 REV	CCC TGA ACC TGG CAA AGG
Band3 -8.6 FWD	TTC AGG GAG GAT AGG TGA GG
Band3 -8.6 REV	CTG CCC TGT CTA AGT ATC TTT CC
Band3 -7.6 FWD	CAT CAC ACT GTT GCT TTC AGC
Band3 -7.6 REV	TTG TCT AAC TCA TCG GGT TTC C
Band3 -6.6 FWD	AGA TGT CCT CAT TGC GTA CC
Band3 -6.6 REV	AGG TAG TCC TAA CTA TTC AAG AAG C
Band3 -5.4 FWD	TGG ATG GTT CAG TGG GTG TAG GG
Band3 -5.4 REV	ACG TGG CTG TAG TTA GCT TGA AGG
Band3 -4.3 FWD	TCT GGA GCA AAG AGT CAC C
Band3 -4.3 REV	CAA ACC CAC TGA ACC AAA GC
Band3 -3.4 FWD	CCT TAC CAC ATC TCT TGT CAC C
Band3 -3.4 REV	GGG TCC TCA AAT CAT CCA TCC
Band3 -2.2 FWD	CGA AGG TCC AGA GTT CAA ATC C
Band3 -2.2 REV	TCT TCA GAC ACT CCA GAA GAG GG
Band3 -0.8 FWD	CTC ATC CCT CCA GGC CAC AGT ACC C
Band3 -0.8 REV	CTG ACT GAC AGA TGA GTT CTG
Band3 Pr FWD	CCC GAG GGT TTG TGA AGG
Band3 Pr REV	CAA AGC ACA TGG ACA CAT TAC C
Band3 +0.8 FWD	GGG TTG ATC ATC TCA GGC TAA C
Band3 +0.8 REV	CTG CCT GCC ACT GAT GAC
Band3 +1.3 FWD	CTG TCC CTG TCA TCT GTC C
Band3 +1.3REV	GA GCT CTG AGT TTG GAA GC
Band3 +2.0 FWD	CCC AGT CCC ACA GTG TTA TTT
Band3 +2.0 REV	CAC CCA GTT TCT CCA GTC TAT C
Band3 +2.8 FWD	GGG TAG GGA CAT CTT TGT CTT G
Band3 +2.8 REV	CAG CCA ACC TGA CTC CAT ATC

Supplementary Table 2. ChIP Primers Continued

Primer	Sequence 5' to 3'
Band3 +3.9 FWD	GGA CTT GGG AAG CCA CTT AAT
Band3 +3.9 REV	CCA GTT CTT CTT CGC TGT CTC
Band3 +4.4 FWD	GCT GGA GAT GCT GAG AAG TGA GAG
Band3 +4.4 REV	GGG CAG TCC AAG GAA AGT GGT ATG
Band3 +6.4 FWD	TTC CTC CAC CAT AGT TCC CAC AAC
Band3 +6.4 REV	CAT GGA CGG AAG AAA GGG TCT CAG
Amylase Pr FWD	TCT TTC TGC TGC TTT CCC TCA T
Amylase Pr REV	CGC TCA CAT TCC TTG GCA ATA TCA

Supplementary Table 2. ChIP Primers Continued

Primer	Sequence 5' to 3'
18S exp FWD	TTG ACG GAA GGG CAC CAC CAG
18S exp REV	GCA CCA CCA CCC ACG GAA TCG
Tspan32 exp FWD	CAC CAT AGC CAC TGT GAG G
Tspan32 exp REV	GGG TTG TAG AAT CTC CAG AAG G
Cd81 exp FWD	GTG GAG GGC TGC ACC AAA T
Cd81 exp REV	GAC GCA ACC ACA GAG CTA CA
Tssc4 exp FWD	AGG AAG AGG GTC CTA AAG AAG G
Tssc4 exp REV	CAA CAG CCT TCA CAC CTA TGG
Gypa exp FWD	TAC CAA GAA GAG CAT TCA CCA TC
Gypa exp REV	TGC TGA TTT GGG TTA CCT ACA GT
Band3 exp FWD	TTC CCA CAG AGC AAA CAG
Band3 exp REV	AGG AAG GTG CCT TTT GAG AAG A

Supplementary Table 3. Expression Primers
