# Supplementary material for: Hyperacetylated chromatin domains mark cell type-specific genes and suggest distinct modes of enhancer function
Source: Nat Commun. 2020 Sep 11;11:4544. doi: 10.1038/s41467-020-18303-0 (PMC7486385; doi:10.1038/s41467-020-18303-0)
Supplement: Supplementary file 3 — Reporting Summary [file 41467_2020_18303_MOESM3_ESM.pdf]

nature research

Corresponding author(s): Bulger, Michael

Last updated by author(s): Apr 23, 2020

Reporting Summary

Nature Research wishes to improve the reproducibility of the work that we publish. This form provides structure for consistency and transparency in reporting. For further information on Nature Research policies, see Authors & Referees and the Editorial Policy Checklist.

Statistics

For all statistical analyses, confirm that the following items are present in the figure legend, table legend, main text, or Methods section.

n/a	Confirmed	
☐	☒	The exact sample size (n) for each experimental group/condition, given as a discrete number and unit of measurement
☐	☒	A statement on whether measurements were taken from distinct samples or whether the same sample was measured repeatedly
☐	☒	The statistical test(s) used AND whether they are one- or two-sided<br>*Only common tests should be described solely by name; describe more complex techniques in the Methods section.*
☒	☐	A description of all covariates tested
☒	☐	A description of any assumptions or corrections, such as tests of normality and adjustment for multiple comparisons
☐	☒	A full description of the statistical parameters including central tendency (e.g. means) or other basic estimates (e.g. regression coefficient) AND variation (e.g. standard deviation) or associated estimates of uncertainty (e.g. confidence intervals)
☐	☒	For null hypothesis testing, the test statistic (e.g. F, t, r) with confidence intervals, effect sizes, degrees of freedom and P value noted<br>*Give P values as exact values whenever suitable.*
☒	☐	For Bayesian analysis, information on the choice of priors and Markov chain Monte Carlo settings
☒	☐	For hierarchical and complex designs, identification of the appropriate level for tests and full reporting of outcomes
☒	☐	Estimates of effect sizes (e.g. Cohen's d, Pearson's r), indicating how they were calculated

Our web collection on statistics for biologists contains articles on many of the points above.

Software and code

Policy information about availability of computer code

Data collection	Illumina HiSeq2500, CFX Connect Real-Time PCR System, CFX Manager
Data analysis	All analysis was done using publicly available tools including bcl2fastq (v1.84.), trimmomatic (v0.32), samtools, bowtie (v1), MACS (v2), bedtools, ROSE, and Enrichr. The exeption to this is our calling of HCDs, for which a custom perl script was written. This script is published along with the manuscript in supplementary data 17.

For manuscripts utilizing custom algorithms or software that are central to the research but not yet described in published literature, software must be made available to editors/reviewers. We strongly encourage code deposition in a community repository (e.g. GitHub). See the Nature Research guidelines for submitting code & software for further information.

Data

Policy information about availability of data

All manuscripts must include a data availability statement. This statement should provide the following information, where applicable:

- Accession codes, unique identifiers, or web links for publicly available datasets
- A list of figures that have associated raw data
- A description of any restrictions on data availability

All data generated for this manuscript has been deposited at GEO with accession: GSE132130 and will be made public upon publication. Additional ChIP-seq, ATAC-seq, and RNA data was downloaded from GEO:
GSE87064: SRR4253101, SRR4253102, SRR5884811, SRR5884812, SRR4252712, SRR4252713, SRR4252826, SRR4252827, SRR4252855, SRR4252856
GSE98724: SRR5520174 SRR55201745, SRR5520168, SRR5520169, SRR5520141, SRR5520142, SRR5520146, SRR5520147
GSE36985: SRR452930, SRR452943, SRR452948, SRR524935
GSM923575: SRR492436, SRR492437, SRR492450, SRR492451, SRR492452
Microarray data was downloaded from ArrayExpression (G_GEOD-36984) and ErythronDB.

Field-specific reporting

Please select the one below that is the best fit for your research. If you are not sure, read the appropriate sections before making your selection.

☒ Life sciences ☐ Behavioural & social sciences ☐ Ecological, evolutionary & environmental sciences

For a reference copy of the document with all sections, see nature.com/documents/nr-reporting-summary-flat.pdf

Life sciences study design

All studies must disclose on these points even when the disclosure is negative.

Sample size	For ChIP-seqs and ATAC-seq n=2 biological replicates taken from different timed matings, with the exception of H3K4Me1 for which n=3. We do not perform differential tests so n=2 was sufficient to define the landscape as well as ENCODE requires a minimum of 2 biological replicates for each antibody. Cost was also a consideration.
Data exclusions	We excluded H1 genes associated with 2 HCDs in the mouse erythroid HCD data set when performing Enrichr cell type enrichment analysis and GO analysis because these are clusters of 10+ genes being called as a domain, not a single gene. This single exclusion was not preestablished, but made during the course of analysis because the inclusion of the histone H1 cluster was dominating the analysis.
Replication	ChIp-seq and ATAC-seq experiments were performed on two biological replicates with the exception of H3K4Me1 which was performed on three biological replicates. ChIP-qPCR and gene expression experiments were performed on three biological replicates with the exception of the Gypa promoter deletion as well as the enhancer replacement experiment where only two biological replicates of the homozygous insertion of the new enhancer were found. Each biological replicate represents the mean of three technical replicates.
Randomization	The experiments described in this study involve assays performed on primary murine erythroid cells only, with no additional treatments or conditions that would require randomization.
Blinding	The experiments described in this study involve assays performed on primary murine erythroid cells derived from fetal liver only, with no additional treatments or conditions. Blinding is therefore superfluous.

Reporting for specific materials, systems and methods

We require information from authors about some types of materials, experimental systems and methods used in many studies. Here, indicate whether each material, system or method listed is relevant to your study. If you are not sure if a list item applies to your research, read the appropriate section before selecting a response.

Materials & experimental systems

n/a	Involved in the study	
☐	☒	Antibodies
☐	☒	Eukaryotic cell lines
☒	☐	Palaeontology
☐	☒	Animals and other organisms
☒	☐	Human research participants
☒	☐	Clinical data

Methods

n/a	Involved in the study	
☐	☒	ChIP-seq
☐	☒	Flow cytometry
☒	☐	MRI-based neuroimaging

Antibodies

Antibodies used	to H3K4Me1 (Abcam #ab8895)<br>H3K4Me3 (Active Motif #39916)<br>H3K27Ac (Active Motif #39134)<br>H3K4Me2 (Abcam #7766))<br>H3K4Me2 (Millipore # 07-030)<br>Nonspecific rabbit immunogloblulin G (Milipore # 12-370)
Validation	Abcam antibodies were validated by the manufacturer by Western blot, ChIP-qPCR, immunohistochemistry, and dot blot; Active Motif antibodies were validated by the manufacturer by Western blot, ChIP-qPCR, immunohistochemistry, and dot blot; Milipore antibodies were validated by the manufacturer by Western blot, ChIP-qPCR, immunohistochemistry, electron microscopy, and dot blot. No validation statements were made.

Eukaryotic cell lines

Policy information about cell lines

Cell line source(s)	MEL cells were used in this study, These cells originate from a subcloning originally performed by Claire Francastel in the lab of Dr. Mark Groudine more than 20 years ago. Aliquots from this source are thawed and used for experiments periodically to this date. The original source of these MEL cells is unclear, except that they were derived (as with all MEL cell lines) by Friend virus infection of mice and isolation of the resulting erythroleukemia cells.
Authentication	MEL cells are a transformed cell line that exhibits aneuploidy. Insofar as authentication is possible, we regularly test proliferating cultures for the characteristics associated with with MEL cells: morphology, rapid (~24 hr, doubling) proliferation, and behavior upon induction of terminal differentiation - as measured by cell/nuclear size, cessation of proliferation, benzidine staining for globin expression and qRT-PCR for induction of expression of late erythroid markers.
Mycoplasma contamination	Cell lines were not tested for mycoplasma contamination.
Commonly misidentified lines (See ICLAC register)	MEL745A are not commonly misidentified given the characteristic behaviors described above.

Animals and other organisms

Policy information about studies involving animals; ARRIVE guidelines recommended for reporting animal research

Laboratory animals	For ChIP-seq and ATAC-seq primary erythroid cells (e14.5 fetal liver) were isolated from <1 year old C57Bl/6J mice via timed matings. Gender was not a consideration. Animal holding rooms are maintained within temperature and humidity ranges described in the ILAR Guide for the Care and Use of Laboratory Animals (1996) - 64-79 degrees F, humidity 30-70%, with occasional (~10 days/year) outliers depending on outdoor seasonal extremes. A 14-hour light/10-hour dark cycle or 12 light/12 dark cycle is commonly used.
Wild animals	This study did not involve wild animals.
Field-collected samples	This study did not involve field-collected samples.
Ethics oversight	The Institutional Animal Care and Use Committee (IACUC) affiliated UCAR at the University of Rochester Medical Center has reviewed and approved all protocols involved in this project for the use of mice.

Note that full information on the approval of the study protocol must also be provided in the manuscript.

ChIP-seq

Data deposition

☒ Confirm that both raw and final processed data have been deposited in a public database such as GEO.

☒ Confirm that you have deposited or provided access to graph files (e.g. BED files) for the called peaks.

Data access links *May remain private before publication.*	GSE132130
Files in database submission	GSM3845584 _H3K4Me1_4-20_peaks.broadPeak.gz<br>GSM3845585_H3K4Me1_4-8_peaks.broadPeak.gz<br>GSM3845586_H3K4Me1_4-1_peaks.broadPeak.gz<br>GSM3845587_ProE-2_peaks.narrowPeak.gz<br>GSM3845588_ProE-1_peaks.narrowPeak.gz<br>GSM3845589_H3K4Me2_3-18_peaks.broadPeak.gz<br>GSM3845590_H3K4Me2_3-25_peaks.broadPeak.gz<br>GSM3845591_H3K4Me3_3-25_peaks.broadPeak.gz<br>GSM3845592_H3K4Me3_3-18_peaks.broadPeak.gz<br>GSM3845593_H3K27ac_3-18_peaks.broadPeak.gz<br>GSM3845595_H3K27ac_3-23_peaks.broadPeak.gz
Genome browser session (e.g. UCSC)	No longer applicable

Methodology

Replicates	All ChIP-seq and ATAC-seq experiments were performed as two independent biological replicates with the exception of H3K4Me1, which was performed as three biological replicates.
Sequencing depth	16-91 million single end reads were generated for all ChIP-seq and ATAC-seq experiments.
Antibodies	to H3K4Me1 (Abcam #ab8895)<br>H3K4Me3 (Active Motif #39916)

	H3K27Ac (Active Motif #39134)<br>H3K4Me2 (Abcam #7766))<br>Nonspecific rabbit immunogloblulin G (Milipore # 12-370)
Peak calling parameters	32 Peaks were called for each replicate of each mark using MACS2 along with additional settings including (--broad-broad-cutoff 0.1 -B) using the total input control as the mock data file.
Data quality	All peaks were retained that passed our quality cutoff of 0.1.
Software	bedtools, ROSE, a custom perl script to identify HCDs that is published along with the manuscript in supplemental data 17.

Flow Cytometry

Plots

Confirm that:

☒ The axis labels state the marker and fluorochrome used (e.g. CD4-FITC).

☒ The axis scales are clearly visible. Include numbers along axes only for bottom left plot of group (a 'group' is an analysis of identical markers).

☒ All plots are contour plots with outliers or pseudocolor plots.

☒ A numerical value for number of cells or percentage (with statistics) is provided.

Methodology

Sample preparation	The cells were resuspended in D-PBS _0.5% FBS(Gemini) and stained with DAPI(ThermoFisher).
Instrument	BD FACSAria II
Software	BD FACSDiva A.0.1
Cell population abundance	For sorts of MEL cells after introduction of CRISPR/Cas9 and sgRNAs (with GFP maker), <1% of the sorted cells were both viable (as assessed by DAPI staining) and GFP positive.
Gating strategy	For GFP+ sorting of MEL cells subsequent to introduction of CRISPR/Cas9 and sgRNAs, single cells were cells were selected by forward and side scatter, live cells selected by DAPI exclusion, GFP positive cells sorted. For ATAC-seq, Proerythroblasts were sorted initially using a broad FSC-A/SSC-A size gate containing ~80% of the cells. Following gating for live cells CD117+ Ter+ double positive cells (1-2%) were further confirmed to be CD44+ (90%), and singlets were obtained by FSCH/W and SSC H/W gating.

☒ Tick this box to confirm that a figure exemplifying the gating strategy is provided in the Supplementary Information.
