# Supplementary material for: Hyperacetylated chromatin domains mark cell type-specific genes and suggest distinct modes of enhancer function
Source: Nat Commun. 2020 Sep 11;11:4544. doi: 10.1038/s41467-020-18303-0 (PMC7486385; doi:10.1038/s41467-020-18303-0)
Supplement: Supplementary file 4 — Description of Additional Supplementary Files [file 41467_2020_18303_MOESM4_ESM.pdf]

Description of Additional Supplementary Files

File Name: Supplementary Data 1
Description: HCD and SE regions identified in mouse erythroid cells.

File Name: Supplementary Data 2
Description: Full Enrichr cell-type enrichment analysis in mouse erythroid cells.

File Name: Supplementary Data 3
Description: Full Enrichr GO analysis in mouse erythroid cells.

File Name: Supplementary Data 4
Description: HCD and SE regions identified in human erythroid cells.

File Name: Supplementary Data 5
Description: Full Enrichr cell-type enrichment analysis in human erythroid cells.

File Name: Supplementary Data 6
Description: Full Enrichr GO analysis in human erythroid cells.

File Name: Supplementary Data 7
Description: HCD and SE regions identified in mouse intestinal epithelial cells.

File Name: Supplementary Data 8
Description: HCD and SE regions identified in mouse retinal cells.

File Name: Supplementary Data 9
Description: Full Enrichr cell-type enrichment analysis in mouse intestinal epithelial cells.

File Name: Supplementary Data 10
Description: Full Enrichr GO analysis in mouse intestinal epithelial cells.

File Name: Supplementary Data 11
Description: Full Enrichr cell-type enrichment analysis in mouse retinal cells.

File Name: Supplementary Data 12
Description: Full Enrichr GO analysis in mouse retinal cells.

File Name: Supplementary Data 13
Description: HCD and Me3 regions identified in mouse erythroid cells.

File Name: Supplementary Data 14
Description: Full HCD vs Me3 Enrichr cell-type enrichment analysis in mouse erythroid cells.

File Name: Supplementary Data 15
Description: Full HCD vs Me3 Enrichr GO analysis in mouse erythroid cells.

File Name: Supplementary Software 1
Description: (.zip file) - identify_HCD readme file and identify_HCD.pl files, comprising the script to call HCDs.
