# Supplementary figures and images for: Hyperacetylated chromatin domains mark cell type-specific genes and suggest distinct modes of enhancer function
Source: Nat Commun. 2020 Sep 11;11:4544. doi: 10.1038/s41467-020-18303-0 (PMC7486385; doi:10.1038/s41467-020-18303-0)

# Hyperacetylated Domains

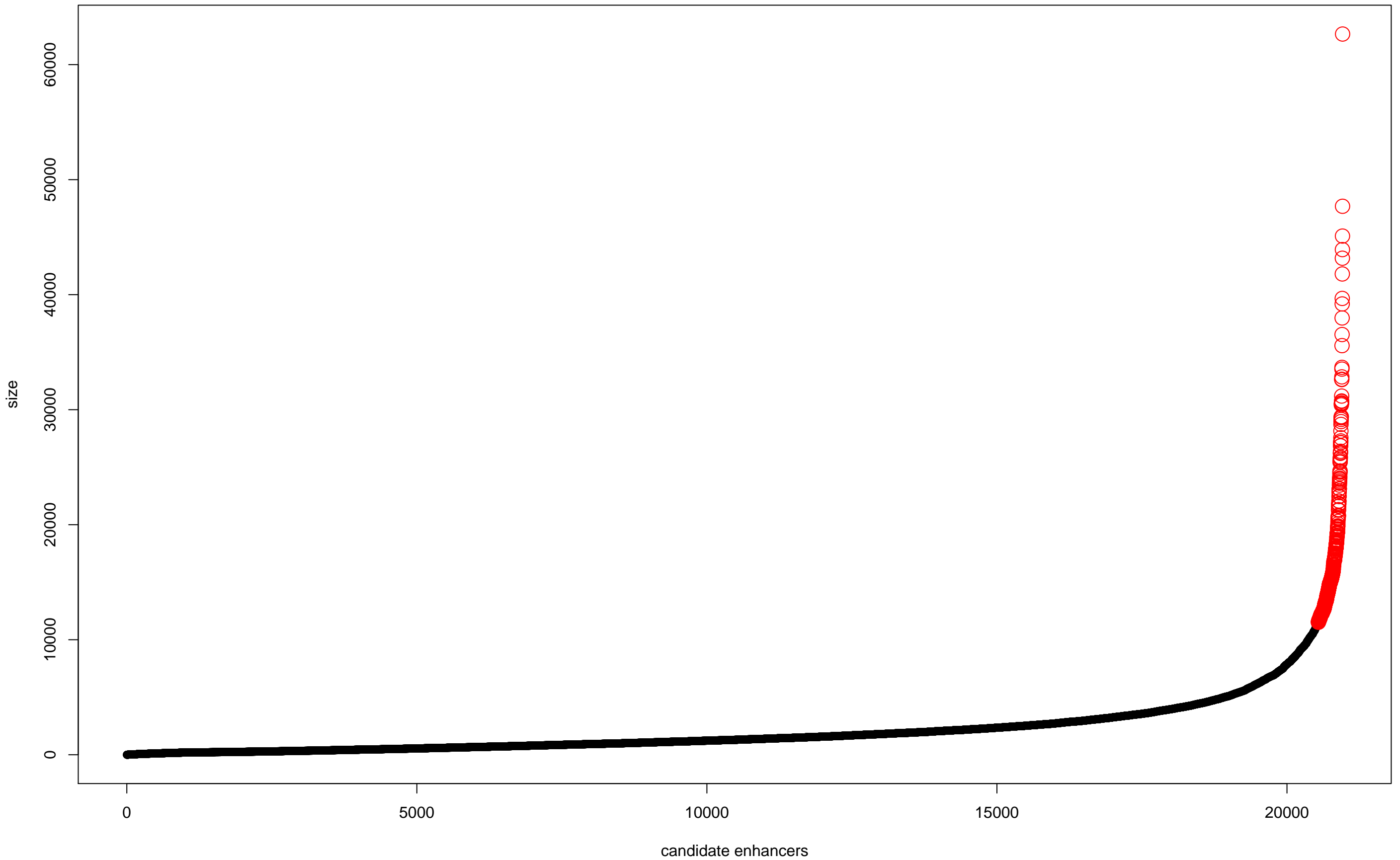

Supplement: Supplementary file 20 — Supplementary Software 1 [file 41467_2020_18303_MOESM20_ESM.zip › identify_HCD/H3K27Ac.pdf]

# Hyperacetylated Domains

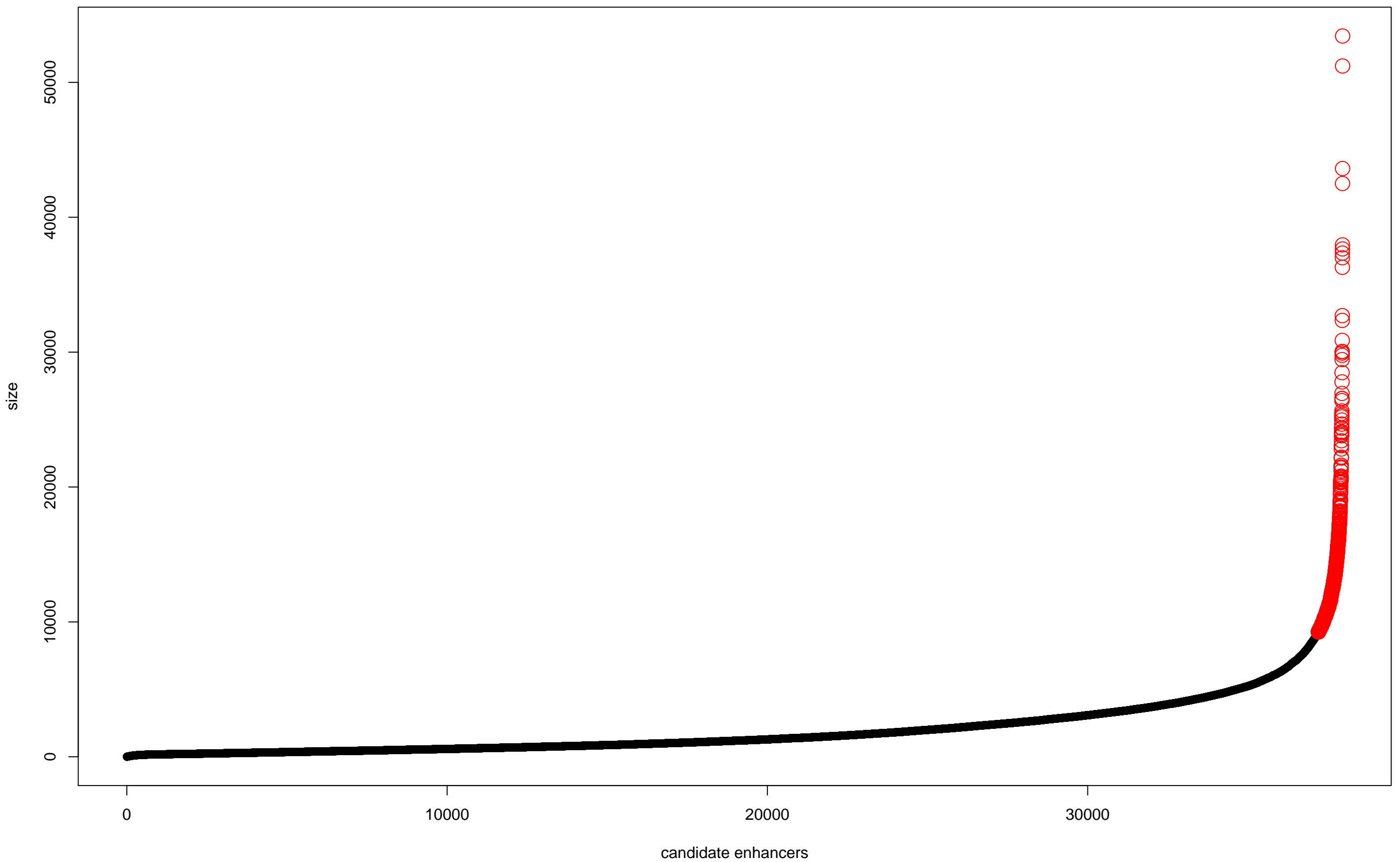

Supplement: Supplementary file 20 — Supplementary Software 1 [file 41467_2020_18303_MOESM20_ESM.zip › identify_HCD/H3K4Me2.pdf]
